# Supplementary material for: SC-JNMF: single-cell clustering integrating multiple quantification methods based on joint non-negative matrix factorization
Source: PeerJ. 2021 Aug 27;9:e12087. doi: 10.7717/peerj.12087 (PMC8404576; doi:10.7717/peerj.12087)
Supplement: Supplemental Information 1 [file peerj-09-12087-s001.pdf]

SUPPLEMENTAL INFORMATION

Rank estimation using loss and sparseness

There has been much discussion on the estimation of rank in NMF; however, no consensus has been reached yet. NMF is evaluated based on the value of the objective function to be minimized (the difference between the original matrix and the reconstructed matrix). However, the optimization problem of NMF is NP-hard, and only its convergence in minimizing the objective function is guaranteed (Vavasis (2009)). Therefore, several different local minima can be obtained using NMF. To mitigate these problems, the factor sparseness in NMF is an important feature, which is sufficient for the uniqueness of the solution, and we consider the sparseness of the factor in the evaluation of the proposed method and the choice of parameters. In this study, the sparseness of the matrix $\boldsymbol{X} \in \mathbb{R}_{+}^{k\times M}$ was formulated as proposed by Hoyer(Hoyer (2004)).

$$\begin{aligned} sp(\boldsymbol{X}) &= \sum_{m} sp(\boldsymbol{x}_{\cdot m}) \\ &= \sum_{m} \frac{\sqrt{K} - (\sum_k |\boldsymbol{x}_{km}|)/\sqrt{\sum_k \boldsymbol{x}_{km}^2}}{\sqrt{K}-1} \end{aligned} \tag{1}$$

where, K is the rank of $\boldsymbol{X}$.

Regarding the optimization of NMF, the lower loss and the higher sparseness(L1/L2 in each vector) were better. Fig. S1 shows the transition of ARI, sparseness, and loss with different ranks in joint-NMF for all datasets.

In general, the loss decreases, and the sparseness increases as the rank increases. Therefore, the rank that stops the decrease in loss or increase in sparseness is optimal because the factorized matrices with larger ranks include information that is not necessary to represent the principal factors of the input matrix. In this experiment, Treutlein Pollen and Xin datasets showed the rank that stopped the increase in sparseness (4–6 in the Treutlein dataset, 7–9 in the Pollen dataset, and 15–30 in the Xin dataset). However, the Segerstolpe dataset showed only a monotonic transition of sparseness and loss. We also used different λ_2 (the regularization parameter for the term on the sparseness of matrix H). The differences in sparseness were confirmed in the Treutlein and Pollen datasets, while the Segerstolpe and Xin datasets showed almost the same sparseness in each rank.

The case of including regularization of the basis matricies

In order to balance the ranges of values between $\boldsymbol{W}$ and $\boldsymbol{H}$, regularization should be imposed not only on the coefficient matrix but also on the basis matrix.

SC-JNMF found the matrix $\boldsymbol{W_1}, \boldsymbol{W_2}, \boldsymbol{H}$ that minimized the following objective function:

$$\begin{aligned} \min_{\boldsymbol{W}_1,\boldsymbol{W}_2,\boldsymbol{H},\lambda_n\geq 0} L := &\|\boldsymbol{D}_1 - \boldsymbol{W}_1\boldsymbol{H}\|_{\mathscr{F}}^2 + \lambda_1\|\boldsymbol{D}_2 - \boldsymbol{W}_2\boldsymbol{H}\|_{\mathscr{F}}^2 \\ &+ \lambda_2 \sum_k \|\boldsymbol{H}^{k,*}\|_1 + \lambda_3 \sum_k \|\boldsymbol{W}_1^{*,k}\|_1 + \lambda_4 \sum_k \|\boldsymbol{W}_2^{*,k}\|_1 \end{aligned} \tag{2}$$

where, λ_2 and λ_3 are parameters for column vector sparsity regularization and λ_4. is a parameter for row vector sparsity regularization.

We applied multiplicative update algorithm to optimize the objective function same as conventional NMF. By applying Jensen's inequalities to the first and second terms of the objective function, the function to be minimized could be rewritten as follows:

$$\begin{aligned} \min_{\boldsymbol{W}_1,\boldsymbol{W}_2,\boldsymbol{H},\lambda_n\geq 0} L := &\sum_{i,j} \left(|\boldsymbol{D}_1^{i,j}|^2 - 2\boldsymbol{D}_1^{i,j} \sum_k \boldsymbol{W}_1^{i,k}\boldsymbol{H}^{k,j} + \sum_k \frac{(\boldsymbol{W}_1^{i,k})^2(\boldsymbol{H}^{k,j})^2}{c_1^{i,j,k}} \right) \\ &+ \lambda_1 \sum_{i,j} \left(|\boldsymbol{D}_2^{i,j}|^2 - 2\boldsymbol{D}_2^{i,j} \sum_k \boldsymbol{W}_2^{i,k}\boldsymbol{H}^{k,j} + \sum_k \frac{(\boldsymbol{W}_2^{i,k})^2(\boldsymbol{H}^{k,j})^2}{c_2^{i,j,k}} \right) \\ &+ \lambda_2\|\boldsymbol{H}\|_1 + \lambda_3\|\boldsymbol{W}_1\|_1 + \lambda_4\|\boldsymbol{W}_2\|_1 \end{aligned} \tag{3}$$

where,

$$c_1^{i,j,k} = \frac{\boldsymbol{W}_1^{i,k}\boldsymbol{H}^{k,j}}{\sum_{k'}\boldsymbol{W}_1^{i,k'}\boldsymbol{H}^{k',j}} \quad (4)$$

$$c_2^{i,j,k} = \frac{\boldsymbol{W}_2^{i,k}\boldsymbol{H}^{k,j}}{\sum_{k'}\boldsymbol{W}_2^{i,k'}\boldsymbol{H}^{k',j}} \quad (5)$$

We found each element of $\boldsymbol{W}_1, \boldsymbol{W}_2$, and $\boldsymbol{H}$ that minimized the objective function by performing partial differentiation.

$$\frac{\partial L}{\partial W_1^{i,k}} = \sum_j (-2D_1^{i,j}H^{k,j} + \frac{2W_1^{i,k}(H^{k,j})^2}{c_1^{i,j,k}}) + \lambda_3 \quad (6)$$

$$\frac{\partial L}{\partial W_2^{i,k}} = \lambda_1 \sum_j (-2D_2^{i,j}H^{k,j} + \frac{2W_2^{i,k}(H^{k,j})^2}{c_2^{i,j,k}}) + \lambda_4 \quad (7)$$

$$\frac{\partial L}{\partial H^{k,j}} = \sum_i (-2D_1^{i,j}W_1^{i,k} + \frac{2(W_1^{i,k})^2H^{k,j}}{c_1^{i,j,k}}) + \lambda_1 \sum_{i'} (-2D_2^{i',j}W_2^{i',k} + \frac{2(W_2^{i',k})^2H^{k,j}}{c_2^{i',j,k}}) + \lambda_2 \quad (8)$$

The objective function are minimized when these are 0. Thus, the variable updates became:

$$\boldsymbol{W}_1 = \boldsymbol{W}_1 \frac{\boldsymbol{D}_1\boldsymbol{H}^\top - \lambda_3/2}{[\boldsymbol{H}[\boldsymbol{W}_1\boldsymbol{H}]^\top]^\top} \quad (9)$$

$$\boldsymbol{W}_2 = \boldsymbol{W}_2 \frac{\lambda_1\boldsymbol{D}_2\boldsymbol{H}^\top - \lambda_4/2}{\lambda_1[\boldsymbol{H}[\boldsymbol{W}_2\boldsymbol{H}]^\top]^\top} \quad (10)$$

$$\boldsymbol{H} = \boldsymbol{H} \frac{[\boldsymbol{D}_1^\top\boldsymbol{W}_1]^\top + \lambda_1[\boldsymbol{D}_2^\top\boldsymbol{W}_2]^\top - \lambda_2/2}{\boldsymbol{W}_1^\top\boldsymbol{W}_1\boldsymbol{H} + \lambda_1\boldsymbol{W}_2^\top\boldsymbol{W}_2\boldsymbol{H}} \quad (11)$$

However, λ_3 and λ_4 did not have a good influence on the clustering in this study; in other words, the L1 norm regularization terms of $|\boldsymbol{W}_1|, |\boldsymbol{W}_2|$ were excessively strong constraints.

REFERENCES

Hoyer, P. O. (2004). Non-negative matrix factorization with sparseness constraints. *Journal of Machine Learning Research*, 5:1457–1469.

Vavasis, S. A. (2009). On the complexity of nonnegative matrix factorization. *SIAM J. on Optimization*, 20(3):1364–1377.
