# Supplementary figures and images for: SC-JNMF: single-cell clustering integrating multiple quantification methods based on joint non-negative matrix factorization
Source: PeerJ. 2021 Aug 27;9:e12087. doi: 10.7717/peerj.12087 (PMC8404576; doi:10.7717/peerj.12087)

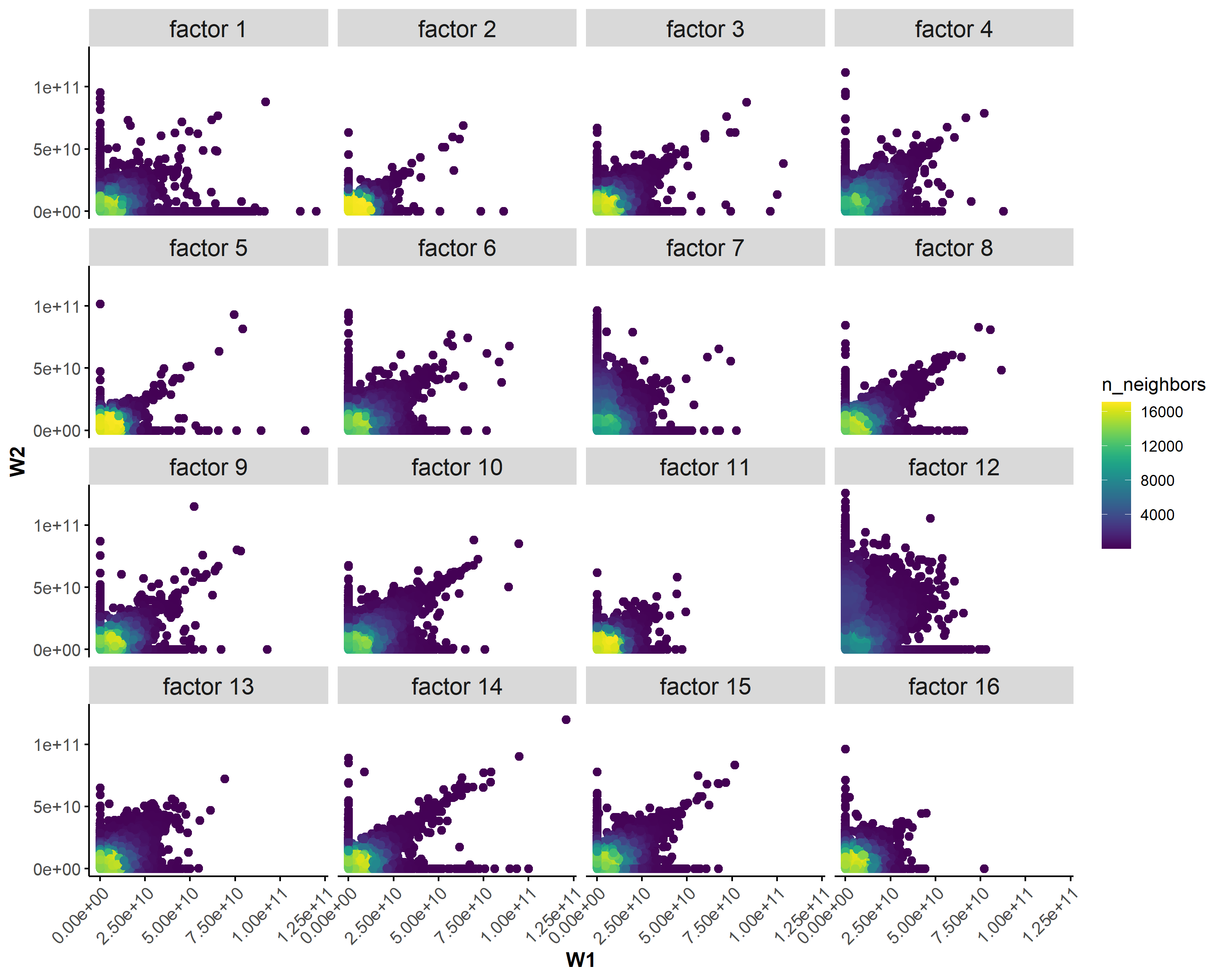

Supplement: Supplemental Information 3 — Points show the genes and their color indicated the density (the number of neighbors). [file peerj-09-12087-s003.png]
